# How does digital governance affect the level of domestic waste separation for rural residents? Empirical evidence from rural areas in Jiangsu Province, China

**DOI:** 10.3389/fpubh.2023.1122705

**Published:** 2023-03-14

**Authors:** Xi Chen, Lirong Xing, Kai Wang, Jianzhen Lu

**Affiliations:** ^1^College of Economics and Management, Nanjing Agricultural University, Nanjing, China; ^2^College of Economics, Shandong University of Technology, Zibo, China; ^3^Institute of Agricultural Economics and Development, Jiangsu Academy of Agricultural Sciences, Nanjing, China

**Keywords:** digital governance, cadre-mass relationship, institutional trust, domestic waste separation, Jiangsu

## Abstract

**Introduction:**

The management of rural domestic waste is directly related to the quality of China's rural habitat and the ecological security of the countryside, and is one of the important tasks of rural revitalization.

**Methods:**

Based on the perspective of digital technology empowering rural governance, this study uses the China Land Economic Survey (CLES) data to empirically test the impact of digital governance on the level of domestic waste separation for rural residents by constructing the ordered probit model.

**Results and discussion:**

The results show that in the process of rural governance modernization, digital governance helps to improve the level of domestic waste separation for rural residents in the process of rural governance modernization, and the findings still hold after robustness tests. Mechanistic tests showed that digital governance can impact the level of domestic waste separation for rural residents through cadre-mass relationship and institutional trust. The findings of this study provide a new perspective on good environmental governance in China's countryside and have important implications for promoting the improvement of rural habitat quality.

## Introduction

Domestic waste management is one of the important initiatives to improve the quality of the rural habitat, and is an inherent requirement for rural revitalization. Currently, China's rural domestic waste production is increasing dramatically at a rate of 8–10% per year (1). The high rate of domestic waste generation creates the dilemma of “garbage-encircled villages,” which not only damages the rural habitat but also seriously threatens the national ecological security. International management experiences have confirmed that the source separation of domestic waste is an important path to effective waste management (2, 3). However, source separation of domestic waste in rural areas in China is currently at a small-scale experimental exploration stage and still faces a serious challenge of domestic waste management. Therefore, the State Council promulgated the *Five-Year Action Program for the Improvement and Upgrading of Rural Habitat (2021–2025)* in December 2021, which includes rural domestic waste management as one of the main ways to improve rural habitat.

In fact, the main obstacle to rural domestic waste management is that rural residents face a collective action dilemma, with low participation rates. From the perspective of economics, there are significant negative externalities associated with the emission of domestic waste from rural residents. To deal with negative externalities, economists have proposed both the Pigouvian and the Coasean paradigms (4). First, the Pigou paradigm, which advocates taxing rural residents for the negative externalities caused by the emission of domestic waste. Second, the Coase paradigm, which advocates clearly defined property rights. However, rural residents are independent individuals with large differences, making it difficult and costly to define property rights. Therefore, such natural resources should be considered public property. According to this logic, the initial right to discharge domestic waste belongs to the government, and rural residents must pay the government to dispose of domestic waste, but they are less environmentally aware and less willing to pay (5). In addition, the Chinese government-led model of rural environmental management is the public-private partnership, which involves a number of entities, such as the government, enterprises and farmers. However, an important problem with this model is that farmers are not sufficiently involved and face problems in organizing collective action. Although the government has adopted economic intervention policies, such as taxes, subsidies and incentives, to promote domestic waste management in rural areas, the sustainability of such policies is limited (6), and they do not provide sufficient incentives for everyone. Combined with the weak regulation of domestic waste segregation in China, it is easy for free rider behavior to occur, resulting in inefficient policy implementation (7). Considering that rural residents are not only the main subjects but also the beneficiaries of rural domestic waste management, their level of participation is directly related to the quality of rural domestic waste management. So, how can rural residents become “endogenous management agents” of domestic waste?

Currently, many scholars have gradually focused on studying the impacting rural residents' domestic waste management. Based on the published literature, the factors can be divided into internal and external factors. The internal factors are mainly based on psychological cognitive perspectives such as environmental concern (8), waste governance cognition (9), environmental governance perceptions (10), and altruistic preferences (11). The external factors are mainly environmental, such as economic incentives (12, 13), transportation conditions (14), fiscal decentralization (8), environmental advocacy, and monitoring (15), and political participation (16). In addition, some scholars have also found that the consistency between rural residents' willingness to separate domestic waste and their separation behavior is low based on the theory of planned behavior (17). From the above literature review, it can be seen that few scholars have examined the impact of digital governance on the management of domestic waste of rural residents based on the digital empowerment perspective.

With the rapid development of digital technologies such as the internet, big data, artificial intelligence, cloud computing and blockchain, they are profoundly influencing and changing the way humans produce, live and govern (18–20). From the perspective of digitally empowered governance approaches, digital governance is a governance model in which the government applies digital technology between the government and residents, enterprises and other subjects, as well as in the internal operation of the government to achieve the purpose of simplifying the process of handling public affairs and achieving democratic governance (21). As the foundation of the national governance system, rural governance is also undergoing a continuous transformation toward digitalization, networking and intelligence (22–24). The Central Internet Information Office and ten other departments jointly issued the *Action Plan for the Development of the Digital Countryside (2022–2025)* in January 2022, which proposes to improve the rural intelligent party building system and promote the extension of “Internet + Government Services” to the countryside (25, 26). It can be seen that as a powerful tool for the government to promote reform and improve governance, digital technology empowerment has changed the culture of rural organization and service delivery, enabling villagers and multiple subjects to participate in the design of public services, and promoting a two-way interactive change in rural social governance (27). This change in governance helps to increase individual participation in village governance practices (28). Currently, digital governance in rural areas focuses on promoting the construction of platforms such as “Internet + Party Building” and “Internet + Government Services” and access to the countryside to recreate and optimize the rural governance space (29). In addition, according to the *Statistical Report on the Development of the Internet in China*, as of December 2020, there were 309 million rural internet users in China, accounting for 31.3% of the total number of internet users, and the rural internet penetration rate was 55.9% (30). This provides a good foundation for digital governance in rural areas of China.

Currently, some scholars have begun to focus on the impact of digital technologies on residents' environmental behavior. In the field of environmental governance, some scholars believe that enhancing environmental information and education has a positive impact on the adoption of environmental behavior, but this impact is moderated by the level of environmental awareness of the population (31). Information tools, such as the internet, have already proven to be a key element in spreading and encouraging environmental programs and improving environmental protection behavior in the home or individuals (32, 33). Most importantly, internet technology can facilitate internet access for vulnerable groups, increase their environmental awareness and promote their participation in source separation of solid waste (34). 83% of women from Kermanshah city in Iran emphasized the role of the internet in increasing their knowledge and attitude toward solid waste recycling (35). 30% of individuals in Kampala city used the internet to access information to improve the level of solid waste management (36). Some scholars also found that using the internet during working hours and using the internet in spare time for learning activities is more conducive to improving individuals' pro-environment behavior (37). Although some scholars have begun to focus on the impact of information technology on individual or family environmental behavior, they have mainly examined the behavior of urban residents. Compared to urban areas, digital governance in rural areas is relatively weak and there is a risk of digital marginalization (38). Then, does digital governance affect the behavior of rural residents in terms of domestic waste separation? If so, what are the mechanisms of this impact? This is worth exploring further.

Compared with previous studies, this study highlights the following aspects. First, few scholars have examined the impact of digital governance on the level of domestic waste separation for rural residents based on the perspective of digital technology empowerment of rural governance. In fact, digital governance is one of the key ways to achieve the modernization of rural governance in China, and the Chinese government is working efforts to implement it. On the other hand, rural domestic waste management is an inherent requirement for improving the quality of rural habitat and realizing the rural revitalization strategy. Therefore, it is necessary for the study to explore the relationship between the two. Second, based on the perspective of cadre-mass relationship and institutional trust, we explore the path of the effect of digital governance on domestic waste separation of rural residents. This study helps to enrich the content of rural residents' domestic waste management behavior, and provides an idea for improving rural habitat management.

This study includes five parts. The first part is an introduction describing the background, problem and significance of the study. The second part proposes the research hypotheses based on relevant theoretical analysis. The third part describes the data and methods used in this study. The empirical analysis and results of the impact of digital governance on the level of domestic waste separation for rural residents are discussed in the fourth part. The fifth part presents the main research conclusions and policy implications.

## Theoretical hypothesis

### Direct impact of digital governance on the level of domestic waste separation for rural residents

First, the information-sharing effect of digital governance. Digital governance has the advantage of information sharing, which can effectively integrate the elements of rural social resources and provide rural residents with the most up-to-date and comprehensive information services (22). The management of domestic waste in rural areas is one of the public affairs of the village. By using the digital governance platform, the policies and regulations on rural domestic waste management and the progress of domestic waste management can be effectively and timely communicated in a timely manner through text, images and video. This way not only improves the publicity of domestic waste management policies, but also increases the awareness and participation of rural residents in domestic waste management policies. In addition, the channels of environmental education for grassroots party members or the public can be widened through the construction of the “Internet + Party Building” platform. In particular, an exemplary role in environmental protection can be played by grassroots party members. Some studies have shown that environmental education can increase the awareness and concern of the rural residents about environmental issues, which in turn promotes their participation in the management of domestic waste (16, 31).

Second, the social monitoring effect of digital governance. As we all know, rural domestic waste management is one of the public affairs of the village, which has the property of quasi-public goods and is very prone to free-riding behavior. This is the root of why rural domestic waste is so difficult to manage. In fact, digital governance mainly facilitates the efficient collection of public opinion by grassroots self-governing organizations and improves the efficiency of two-way communication between the government and the public (19, 39), which has a public participation and monitoring function (40). In other words, digital governance not only helps to achieve mutual supervision between rural residents and grassroots cadres in rural domestic waste management issues, but also provides a diversified channel for rural residents to participate in the public affairs of village domestic waste management. At present, online supervision is becoming more common and has the potential to support supervision in rural areas (41). In rural areas of China, some provinces, such as Zhejiang and Hunan, have started to implement the “Internet + supervision” model. Kathuria's (42) study confirms that public participation in social monitoring can improve the level of environmental regulation. In addition, the externalities of social monitoring can also lead to positive responses from actors, which manifest themselves as an internal drive (43). This reduces the likelihood of rural residents free-riding on domestic waste management and increases their participation in domestic waste management (44). In short, in the area of domestic waste management, digital governance strengthens the mutual supervision between rural residents and village cadres. Village cadres pay more attention to rural domestic waste separation due to the environmental protection performance evaluation by higher government departments. Rural residents, who are bound by supervision and the increased risk of punishment for bad behavior such as littering, will actively participate in the separation of domestic waste out of rational choice. Based on the above analysis, we propose the following hypothesis:

**H**_**1**_. Digital governance helps to improve the level of domestic waste separation for rural residents.

### Mediating effect of cadre-mass relationship and institutional trust

Digital governance, with the advantage of information sharing, helps to eliminate hierarchical boundaries in the cadre-mass relationship, making the relationship between subjects fair and democratic (45). This helps to enhance mutual understanding and trust between villagers and village cadres, forming a good cadre-mass relationship. Cadre-mass relationship is a special form of social network, an important component of social capital, and plays an important role in the work of rural society (46). According to the embeddedness theory perspective the social network in which an individual lives influences his or her behavioral decisions (47). In rural China, which has been a typical acquaintance society since ancient times, social networks are formed through kinship, blood and local ties, and cultures such as human kindness and face(*mianzi*) are deeply rooted in the soil of the vernacular social network and have an important influence on the functioning of the rural social ecology (48). It was shown that social networks can increase rural residents' familiarity, reduce uncertainty in making behavioral decisions, and increase their level of domestic waste separation (49–51). In addition, the social network formed by the cadre-mass relationship can also enhance the flow and exchange of information and increase the level of trust among each other, thus promoting the participation of rural residents in rural environmental governance (52). Based on the above analysis, we propose the following hypothesis:

**H**_**2**_. Digital governance can improve the level of domestic waste separation for rural residents by promoting cadre-mass relationship.

Information visualization theory suggests that the visual presentation of information through graphics and video enhances the reception of information by individuals (53). With the advantage of digitalization, the rural digital platform expands the publicity channels of the domestic waste management policy and effectively realizes the combination of text and image or video presentation. This helps to increase rural residents' awareness of the rural domestic waste management policy, which builds institutional trust. Institutional trust refers to citizens' trust in government and political institutions (54), and is an important measure of the health of democracy in modern countries (55, 56). From a theoretical perspective, behavioral public management suggests that institutional trust is a heuristic tool to stimulate citizen participation and support government action (57, 58). Moreover, institutional trust can also create a soft constraints that can effectively discourage opportunistic behavior such as free-riding and avoid the prisoner's dilemma (59, 60). Binding mechanisms based on institutional trust can be effective in reducing the cost of implementing policies and increasing rural residents' awareness of environmental institutional policies (61). Some scholars have also found that institutional trust can improve rural residents' participation in rural environmental governance (62, 63). Based on the above analysis, we propose the following hypothesis:

**H**_**3**_. Digital governance can improve the level of domestic waste separation for rural residents by promoting institutional trust.

**Figure 1:**
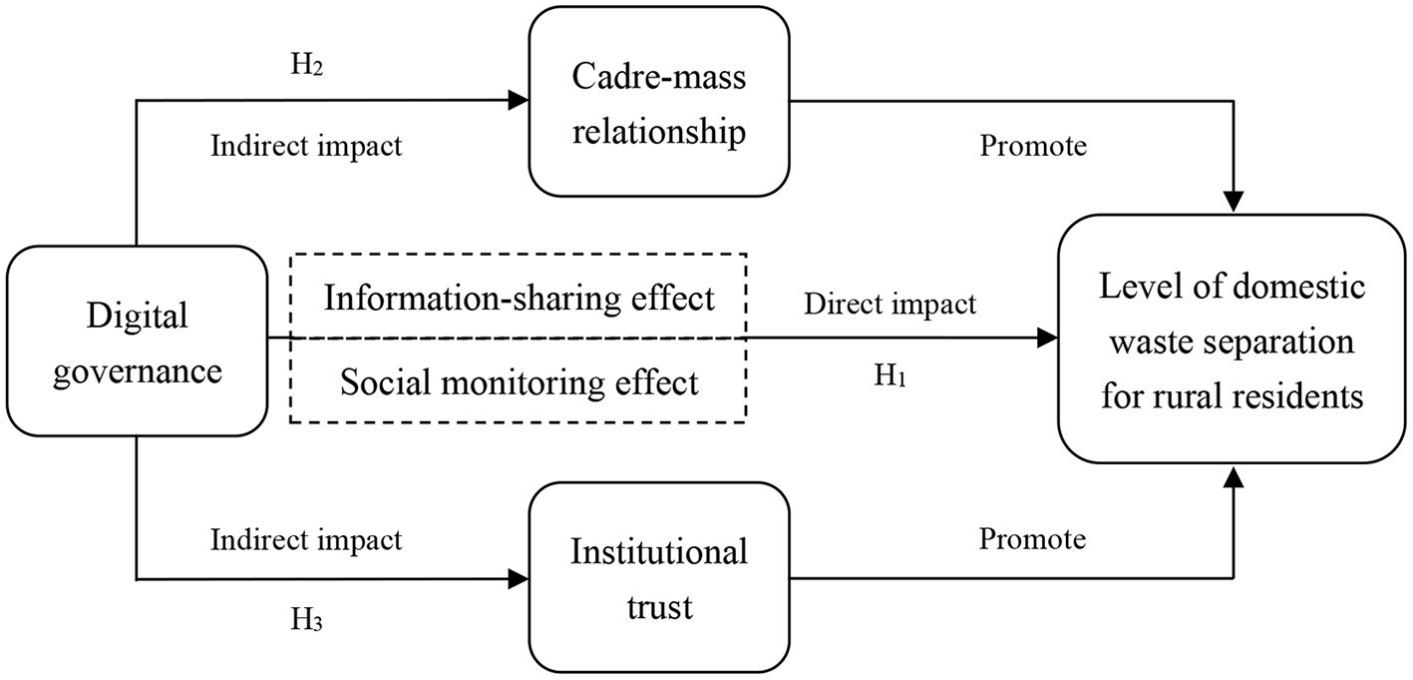
Mechanisms of the impact of digital governance on the level of domestic waste separation for rural residents.

Based on the above analysis, this study proposes a mechanism map of the impact of digital governance on the level of domestic waste separation for rural residents (Figure 1).

## Data and methods

### Data sources

Data from the China Land Economy Survey (CLES) conducted by Nanjing Agricultural University in 2021. The survey covered rural areas in Jiangsu Province. The research questionnaire consists of a questionnaire for rural residents and a questionnaire for villages. The rural resident questionnaire covers rural resident production behavior, land use, family income and expenditure and assets, rural finance, rural governance, etc. The village questionnaire covers the demographic characteristics of the village, the land situation, the collective economy and the external environment. The research activity uses PPS sampling, for each prefecture-level city in Jiangsu Province, two counties and districts are randomly selected, each county and district is randomly selected two townships, each township is randomly selected one administrative village, each village is selected 50 households, the total sample of 2627 households. After removing samples with missing values and outliers from the questionnaire, the final sample size was 2342.

### Variable descriptions

#### Dependent variable

The dependent variable in this paper is the level of domestic waste separation (DWS) for rural residents. Based on the research experience of relevant scholars (64), and combining the current domestic waste classification standards and the living habits of rural residents, the level of domestic waste separation for rural residents is set at four levels, as shown in Table 1. Among the 2,339 rural residents surveyed, 51.56% of rural residents had not yet separated their domestic waste, 32.66% had separated their domestic waste into two categories, 11.37% into three categories and 4.40% into four categories.

**Table 1:** Statistics on the level of domestic waste separation for rural residents.

**Question**	**The level of domestic waste separation for rural residents**	**Number of samples (*N*)**	**Frequency (%)**
How do you dispose of your domestic waste?	1 = Uncategorized (Dump all your waste together)	1,206	51.56
	2 = Two categories (Recyclable/sellable for money, others)	764	32.66
	3 = Three categories (Recyclable/sellable for money, food waste/putrescible, other)	266	11.37
	4 = Four categories (Recyclable/sellable for money, food waste/putrescible, toxic and hazardous, other)	103	4.40

#### Core independent variable

The core independent variable in this paper is the level of digital governance (DG). The current forms of digital governance mainly include “Internet + Party Building,” “Internet +Public Services,” etc. The Central Internet Information Office and ten other departments jointly issued the *Action Plan for the Development of Digital Villages (2022–2025)* and the *Implementation Opinions on Promoting the Construction of High-Quality Digital Villages* issued by Jiangsu Province, both of which set out the requirements for extending “Internet + Party Building” and “Internet + Government Services” to the countryside. The connotation of “Internet + Party Building” is to promote the disclosure of various kinds of information, widen the communication channels between the Party and the masses, and smooth the flow of public opinion. The “Internet + Government Services” emphasizes the sharing of government information and the online processing of agriculture-related matters. Building a digital governance platform and connecting it to the countryside is the basis for implementing digital governance. At present, the sinking of digital governance platforms into the countryside is the key to improving digital governance in rural areas, and is an inevitable requirement for narrowing the digital divide between urban and rural areas. Therefore, this paper uses the connectivity of digital governance platforms in villages to measure the level of digital governance in rural areas. In the CLES village questionnaire, village council members were asked about the digital governance of their village. If the sample village is connected to both the “Internet + party building” and “Internet + government services” platforms, the level of digital governance is assigned a value of 2. If only one of these platforms is connected, the value is assigned to 1. If the sample village is not connected to the “Internet + Party Building” and “Internet + Government Services” platforms, the level of digital governance is assigned a value of 0.

#### Mediated variables

As explained above, the mediating variables in this study include cadre-mass relationship (CMR) and institutional trust (INT). First, cadre-mass relationship is a special type of social capital that can stimulate the endogenous power of villages and fully mobilize villagers to participate in rural governance. Among them, cadre-mass relationship includes both the villagers' trust in village cadres (VTVC) and village cadres' attitude toward serving the public (65). The CLES rural residents questionnaire asked respondents about their level of trust in village cadres, with a range of values from 1 to 5, with higher values indicating a higher level of trust in village cadres. In addition, the CLES rural residents questionnaire also asked respondents about the service attitude of village party cadres (SAVPC) in working for the people. The specific content of the questionnaire is “In the process of contact with village party cadres, how do you feel their work attitude toward serving the public?”. The range of values is 1 to 4, namely “cold attitude and poor service,” “lack of patience and average service,” “average attitude and service,” “enthusiastic and good service.”

Second, institutional trust. Based on the CLES rural residents questionnaire, institutional trust is measured by the “level of trust in the laws and regulations governing domestic waste management in the village.” This is measured on a 5-point Likert scale from “not at all” to “a great deal of trust.” The higher the score, the more rural residents trust domestic waste management system in their village. The statistics of the CLES survey sample show that the mean value of institutional trust reached 3.878, which shows that rural residents trust the laws and regulations related to domestic waste management implemented in their villages. This is closely related to the efforts of the grassroots government's efforts in recent years, which have led to improvements in the rural habitat and increased the level of institutional trust for rural residents.

Control variables. The factors affecting the level of domestic waste separation for rural residents are complex. To reduce omitted variable bias and improve the accuracy of the estimation results, this study controls for the individual characteristics, household characteristics and village characteristics of the respondents, respectively.

#### Individual characteristics

It includes: “age of the respondents (Age)”; “gender of the respondents (Gender),” male = 1, female = 0; “years of education of the respondents (Education),” illiterate = 0, primary school = 6, middle school = 9, high school =12, technical secondary school/vocational high school = 14, college=15, bachelor = 16, master = 19, doctorate = 22; “health of the respondents (Health),” incapacitated = 1, poor = 2, moderate = 3, good = 4, excellent = 5.

#### Household characteristics

Family income (FAI) refers to total household income (CNY 10000). Family size (FAS) is expressed as the number of permanent residents in the family. Family political belief (FPB) is expressed by whether there are party members in the family. If there are party members in the family, a value of 1 was assigned, otherwise, 0.

#### Village characteristics

Village location (VIL) is expressed by the distance between the village and the township government (km). Village economy (VIE) is indicated by whether the village is economically weak or not. If the village economy is weak, a value of 1 was assigned, otherwise, 0. Village sanitation facilities (VSF) are expressed by the number of village garbage cans (bins). Village income per person (VIPP) is expressed in survey specific values (CNY 10,000/person). Table 2 shows the description and statistics of the relevant variables.

**Table 2:** Variable descriptive statistics.

**Indicator**	**Variable**	**Mean**	**Std. dev**.	**Min**	**Max**
Level of domestic waste separation	LDWS	1.686	0.841	1	4
Digital governance	DG	1.429	0.801	0	2
Villagers' trust in village cadres	VTVC	4.069	0.824	1	5
Service attitude of village party cadres	SAVPC	3.735	0.543	1	4
Institutional trust	INT	3.878	0.916	1	5
Gender of the respondents	Gender	0.924	0.264	0	1
Age of the respondents	Age	63.336	10.598	14	98
Education of the respondents	Education	7.310	3.653	0	20
Health of the respondents	Health	4.012	1.093	1	5
Family income	FAI	3.069	11.737	0	303.5
Family size	FAS	3.039	1.584	0	9
Family political belief	FPB	0.310	0.463	0	1
Village location	VIL	6.105	5.976	1	40
Village economy	VIE	0.390	0.488	0	1
Village sanitation facility	VSF	518.050	875.972	3	4,636
Village income per person	VIPP	2.431	1.050	0.236	5

### Model

The level of domestic waste separation for rural residents is an ordered discrete variable. Therefore, we use the ordered probit model to analyze the impact of digital governance on it. The model form is as follows:


(1) $$\begin{array}{l} Dwc^{*}=\alpha Dg+\gamma Control+\varepsilon \end{array}$$

In formula (1), Dwc^*^is an unobservable latent variable. *Dg* represents the level of digital governance. *Control*_*it*_ is a control variable that includes individual characteristics, household characteristics and village characteristics. α and γ are the coefficients of the independent variable. ε_*it*_ is a random error term. Selection models for *Dwc* can be constructed using latent variables. The specific form of the model is as follows:


(2) $$\begin{array}{l} \begin{array}{l} Dwc=\left\{\begin{array}{l} 1,\;Dwc^{*}\leq {\text{λ}}_{1}\\ 2,\;{\text{λ}}_{1}<Dwc^{*}\leq {\text{λ}}_{2}\\ 3,\;{\text{λ}}_{2}<Dwc^{*}\leq {\text{λ}}_{3}\\ 4,\;{\text{λ}}_{3}<Dwc^{*} \end{array}\right\} \end{array} \end{array}$$

In formula (2), *Dwc* represents the level of domestic waste separation for rural residents, and is an ordered variable from 1 to 4. λ_1_ < λ_2_ < λ_3_ is the parameter to be estimated, which is 3 cut points, classifying the level of domestic waste separation of rural residents into 4 levels of hierarchy. When *Dwc*=1, it means that rural residents do not separate their domestic waste. When *Dwc*=2, it means that rural residents divide their domestic waste into two categories. When *Dwc* = 3, it means that rural residents divide their domestic waste into three categories. When *Dwc* = 4, it means that rural residents divide their domestic waste into four categories.

## Empirical results analysis

### Regression results for direct effects

First, a multicollinearity test is carried out. The results showed that the variance inflation factor (VIF) of each explanatory variable took values between 1.03 and 1.45, the mean value of which was 1.17, proving that there is no problem of multicollinearity in the regression model. Second, in order to eliminate the effects of heteroskedasticity and autocorrelation of the residuals on the model estimation results, all regressions in this paper were estimated using clustering robust standard errors (clustering to the village level). Finally, to ensure the quality of the regression results, the individual characteristics, household characteristics, and village characteristics of the respondents were progressively introduced into the ordered probit model.

The estimation results in Table 3 show that the Pseudo *R*^2^ of model M4 is 0.044, which is greater than the results of model M1–model M3, after including the level of digital governance, the level of domestic waste separation of rural residents and the control variables in the model at the same time. This suggests that the estimation results of model M4 are more suitable for research analysis. To further illustrate the impact of digital governance on the level of domestic waste separation for rural residents, the marginal effects are reported based on model M4 (Table 4). Next, the paper will focus on Table 4 for a more detailed analysis.

**Table 3:** Regression results on the direct impact of digital governance on the level of domestic waste separation for rural residents.

**Variables**	**Level of domestic waste separation (LDWS)**			
**M1**	**M2**	**M3**	**M4**
DG	0.136^***^	0.131^***^	0.118^***^	0.136^***^
	(0.031)	(0.031)	(0.031)	(0.035)
Gender		0.036	0.024	0.098
		(0.090)	(0.090)	(0.090)
Age		−0.012^***^	−0.012^***^	−0.013^***^
		(0.003)	(0.003)	(0.003)
Education		0.023^***^	0.019^**^	0.016^**^
		(0.007)	(0.008)	(0.008)
Health		0.061^**^	0.055^**^	0.029
		(0.024)	(0.024)	(0.025)
FAI			0.007^***^	0.006^***^
			(0.001)	(0.001)
FAS			0.055^***^	0.050^***^
			(0.015)	(0.016)
FPB			0.106^**^	0.048
			(0.053)	(0.055)
VIL				−0.004
				(0.004)
VIE				0.061
				(0.063)
VSF				1.6e-04^***^
				(2.29e-05)
VIPP				0.146^***^
				(0.026)
Log likelihood	−2495.069	−2421.193	−2404.530	−2263.405
Wald	19.720^***^	93.320^***^	134.440^***^	248.310^***^
Pseudo *R*^2^	0.004	0.020	0.025	0.044
Observations	2,288	2,257	2,256	2,175

^*^, ^**^, and ^***^ indicate significance at the 10%, 5%, and 1% levels, respectively. Robust standard errors are in brackets.

**Table 4:** Marginal effects of the ordered probit model M4.

**Variables**	**Level of domestic waste separation (LDWS)**			
**level 1**	**level 2**	**level 3**	**level 4**
DG	−0.051^***^	0.021^***^	0.019^***^	0.011^***^
	(0.013)	(0.005)	(0.005)	(0.003)
Gender	−0.037	0.015	0.014	0.008
	(0.034)	(0.014)	(0.013)	(0.008)
Age	0.005^***^	−0.002^***^	−0.002^***^	−0.001^***^
	(0.001)	(0.000)	(0.000)	(0.000)
Education	−0.006^**^	0.002^**^	0.002^**^	0.001^**^
	(0.003)	(0.001)	(0.001)	(0.001)
Health	−0.011	0.004	0.004	0.002
	(0.009)	(0.004)	(0.003)	(0.002)
FAI	−0.002^***^	0.001^***^	0.001^***^	4.827e-04^***^
	(0.000)	(0.000)	(0.000)	(0.000)
FAS	−0.019^***^	0.008^***^	0.007^***^	0.004^***^
	(0.006)	(0.002)	(0.002)	(0.001)
FPB	−0.018	0.007	0.007	0.004
	(0.021)	(0.008)	(0.008)	(0.005)
VIL	0.001	−0.001	−0.001	−3.03e-04
	(0.001)	(0.001)	(0.001)	(0.000)
VIE	−0.022	0.009	0.009	0.005
	(0.024)	(0.010)	(0.009)	(0.005)
VSF	−6.02e-05^***^	2.44e-05^***^	2.24e-05^***^	1.33e-05^***^
	(8.51e-06)	(3.77e-06)	(3.26e-06)	(2.17e-06)
VIPP	−0.055^***^	0.022^***^	0.020^***^	0.012^***^
	(0.009)	(0.004)	(0.004)	(0.002)

^*^, ^**^, and ^***^ indicate significance at the 10%, 5%, and 1% levels, respectively. Robust standard errors are in brackets.

Second, the impact of digital governance. Model M4 estimation results in Table 3 show that digital governance has a positive relationship with the level of domestic waste separation for rural residents. This indicates that the higher the level of digital governance, the higher the level of domestic waste separation for rural residents. The marginal effects in Table 4 show that for each unit increase in the level of digital governance, the probability of rural residents not separating their domestic waste decreases by 5.1% and the probability of separating their domestic waste into two, three and four categories improve by 2.1, 1.9, and 1.1%, respectively. A relatively reasonable explanation is that digital governance has promoted the dissemination of national policies on rural domestic waste management, broadened rural residents' access to information about domestic waste management and raised their cognition of the hazards of domestic waste and their awareness of separation. However, digital governance is still at a preliminary stage, and the acquisition of knowledge and awareness of domestic waste separation by rural residents is a process that is constantly deepening. Therefore, its impact on rural residents' domestic waste separation shows a decreasing effect from simple to complex separation. In other words, its influence has a ripple effect. Thus, it is clear that digital governance helps to promote the level of domestic waste separation for rural residents. Therefore, hypothesis H_1_ is confirmed.

Finally, the impact of control variables was analyzed. The age of the respondents was significantly negative at the 1% level, indicating that the older the rural residents were, the lower the level of domestic waste separation. A plausible explanation is that the older the rural residents are, the more their awareness and knowledge of domestic waste separation is relatively low, leading to a lower level of domestic waste separation. The number of years of education of the respondents was significantly positive at the 5% level, indicating that the longer the years of education of the rural residents, the higher the level of domestic waste separation. It is possible that education can make rural residents aware of the importance and necessity of waste separation. Both family income (FAI) and village income per person (VIPP) were significantly positive at the 1% level, indicating that higher income levels help to improve the level of domestic waste separation for rural residents. As the Chinese proverb says, “When the granaries are full, people follow appropriate rules of conduct, and when there is enough to eat and wear, people know honor and shame.” Family size (FAS) was significantly positive effect at the 1% level. It may be that the more people there are in a household, the more waste there is. If the waste is not separated and disposed of in time, it will not only affect the environment around the house but also endanger the health of the family. The more complete the village sanitation facilities (VSF), the higher the level of domestic waste separation for rural residents. A possible explanation is that VSF not only reduces the cost of domestic waste management for rural residents to a certain extent, but also provides convenience for rural residents to separate their domestic waste.

### Mediation effect test

The findings above confirm hypothesis H_1_, which states that digital governance has a significant positive effect on the level of separation of domestic waste for rural residents. Next, we test hypothesis H_2_ and hypothesis H_3_ to analyze why digital governance has an impact on the level of domestic waste separation for rural residents. This will help to summarize the current issues and provide empirical revelations of rural domestic waste separation.

First, Model M1 and Model M2 in Table 5 test the impact of digital governance on the cadre-mass relationship (CMR). According to the regression results of the ordered probit model, digital governance has a significant positive effect on the villagers' trust in village cadres (VTVC) and the service attitude of village party cadres (SAVPC), indicating that digital governance helps to promote cadre-mass relationship. Therefore, digital governance can improve the level of domestic waste separation for rural residents by promoting cadre-mass relationship.

**Table 5:** Regression results for impact mechanisms.

**Variables**	**Cadre-mass relationship (CMR)**		**Institutional trust (INT)**
	**VTVC**	**SAVPC**	
	**M1**	**M2**	**M3**
DG	0.061^*^	0.096^**^	0.060^*^
	(0.032)	(0.039)	(0.033)
Control variables	Yes	Yes	Yes
Log likelihood	−2488.308	−1360.451	−2569.498
Wald	74.410^***^	73.550^***^	77.560^***^
Pseudo *R*^2^	0.015	0.025	0.015
Observations	2,174	2,169	2,064

Note: ^*^, ^**^, and ^***^ indicate significance at the 10%, 5%, and 1% levels, respectively. Robust standard errors are in brackets.

Second, Model M3 in Table 5 tests the impact of digital governance on institutional trust. According to the regression results of the ordered probit model, digital governance has a significant positive effect on institutional trust. This indicates that digital governance can improve the level of domestic waste separation for rural residents by promoting institutional trust. Therefore, hypothesis H_2_ and hypothesis H_3_ are confirmed.

### Robustness test

(1) Endogeneity issues. There may be some endogeneity issues with the above regressions, which may lead to inconsistent estimation results. Therefore, the regressions were re-estimated using the instrumental variables method. The level of digital cognition of village cadres (DCVC) was chosen as the instrumental variable in this study. Two questions were addressed in the CLES village questionnaire. First, do you think it is necessary to adopt the form of “Internet+” (e.g. “Internet + community”) to promote rural governance? Second, do you think it is necessary to improve the digital literacy of farmers? Each is measured on a 5-point Likert scale from “totally unnecessary” to “very necessary.” The above two questions reflect the level of awareness of digital transformation for village cadres from the perspective of digital literacy of the providers and participants of digital governance, respectively. In this study, the responses to the above two questions will be added together to obtain the level of DCVC.

There are two main reasons for choosing DCVC as an instrumental variable in this study. First, village cadres are the practitioners of digital governance in rural areas. In rural governance, village cadres are the foundation of the government's work in rural areas, and are the leaders in improving the capacity of grassroots governance and the practitioners of governance modernization. It can be said that the construction of a digital platform for villages is closely related to village cadres. Second, cognition determines behavior. The theory of planned behavior (TPB) generally suggests that individuals' behavioral intentions are influenced to some extent by their level of cognition. In other words, the stronger the digital cognition of village cadres, the more they will help to promote the digital transformation of rural governance. However, the personal digital cognition of village cadres does not directly impact the level of domestic waste separation of other villagers. Therefore, the instrumental variables chosen in this paper theoretically meet the requirements of relevance and exogeneity.

Next, econometric methods are used to further test the appropriateness of the instrumental variables chosen for this paper. The Durbin-Wu-Hausman (DWH) test showed that the values corresponding to Durbin (score) chi2 and Wu-Hausman F were 21.839 and 21.919, respectively, both significant at the 1% level, making it necessary to use the instrumental variable method. The under-identification test showed a Kleibergen-Paap rk LM statistic of 139.397, rejecting the original hypothesis of under-identification at the 1% level of significance. The weak instrumental variable test showed that the Kleibergen-Paap rk Wald F statistic of 405.788 was much greater than the critical value of 16.38 at the 10% level of the Stock-Yogo test, rejecting the original hypothesis that the selected instrumental variable was a weak instrumental variable. It can be seen that the instrumental variables selected for this study are relatively appropriate.

Given that the dependent variable is an ordered discrete variable, the conditional mixed process (CMP) method was used for estimation to address the endogeneity of the model. The CMP method is a two (multi) stage regression model that is based on a seemingly uncorrelated regression, estimated using the method of great likelihood, and implemented by constructing a recursive system of equations (66). The results of the estimation of model M1 in Table 6 show that the DCVC is significant at the 1% level and satisfies the correlation, and the atanhrho_12 value also passes the significance test, indicating that the CMP estimation is more appropriate at this point. After using instrumental variables to address endogeneity, the effect of digital governance on the level of domestic waste separation for rural residents rises to 0.714, which is larger than the estimated coefficient of the ordered probit regression (model M4 in Table 3), indicating that the ordered probit estimates above are somewhat biased downwards. This suggests that the estimation results in this paper are robust.

**Table 6:** Estimation results of robustness tests.

**Variables**	**CMP method**		**Ordered logit model**	
	**M1**		**M2**	
	**Coefficient**	**Standard error**	**Coefficient**	**Standard error**
DG	0.714^***^	0.103	0.227^***^	0.061
DCVC	0.143^***^	0.007		
atanhrho_12	−0.483^***^	0.094		
Control variables	Yes	Yes	Yes	Yes
Log likelihood	−4546.853		−2261.190	
Wald	1869.280^***^		252.930^***^	
Pseudo *R*^2^			0.045	
Observations	2,178		2,175	

Note: ^*^, ^**^, and ^***^ indicate significance at the 10%, 5%, and 1% levels, respectively. Robust standard errors are in brackets.

(2) Replacement model testing. To further test the reliability of the model estimation results, digital governance, the level of domestic waste separation for rural residents and control variables were introduced into the ordered logit model for regression. The estimation results of model M2 in Table 6 show that the impact of digital governance on the level of domestic waste separation by rural residents is significant in line with the estimation results of model M4 in Table 3, with only the regression coefficients differing. This suggests that the estimation results in this paper are robust.

## Conclusions and policy implications

To implement rural revitalization and build a beautiful countryside, “green” is the “base color.” As an important pollution prevention and treatment project to improve the quality of rural habitat, the effective management of rural domestic waste cannot be achieved without the active participation of rural residents. So, how can we increase the level of domestic waste separation for rural residents? Based on digital technology empowering rural governance perspective, this study uses CLES survey data to empirically explore the impact of digital governance on the level of domestic waste separation for rural residents by constructing the ordered probit model. The study found that digital governance helps to improve the level of domestic waste separation for rural residents in the process of modernizing rural governance, and the findings still hold after robustness tests. Mechanistic tests showed that digital governance can impact the level of domestic waste separation for rural residents through cadre-mass relationship and institutional trust.

Based on the above conclusions, the following policy recommendations are proposed.

(1) Continue to strengthen digital governance in rural areas and improve the level of domestic waste separation for rural residents. In the process of modernizing rural governance, it is important to continuously strengthen rural informatization and embed digital technology into rural governance. On the one hand, government departments need to accelerate the extension of digital platforms to rural areas and increase the depth and breadth of digital governance applications in the rural sector. On the other hand, through the digital governance platform, an environmental protection information column will be opened to publish information on the management of rural domestic waste, and village cadres will be asked to lead rural residents to learn and understand about waste management together. The main objective is to raise the level of awareness of village cadres and rural residents on domestic waste management.

(2) Establishing good cadre-mass relations and helping to implement rural environmental management on the ground. Asking the people not only strengthens the village cadres' understanding of the problems that exist in the process of rural domestic waste management, but also enables them to contact and care for the villagers, understand their ideas and needs in domestic waste management, and truly implement the policy on the ground. Therefore, in rural environmental management, village cadres should strengthen their liaison and communication with villagers more often and adopt a combination of online and offline methods, such as holding seminars, democratic life meetings, regular visits, etc. Especially in the current period of high prevalence of infectious diseases, such as COVID-19, it is important to take advantage of digital governance. Through digital platforms such as “Internet + Party Building” and “Internet + Government Services,” it is possible not only to expand the communication channels between the Party and the masses, but also to publicize the policies and effectiveness of domestic waste management public through digital platforms in a timely manner. The aim is to build a good image and credibility of open village affairs, establish a good cadre-mass relationship, and reduce the cost of rural environmental management.

(3) Improve the policy system of rural domestic waste management and enhance the participation of rural residents. Government departments should adopt a fair and democratic approach to guide rural residents to participate in the formulation, implementation and management of rural domestic waste policies. Through democratic discussions and other means, rural residents will be encouraged to meet face-to-face with policy makers and implementers to interpret and improve existing policies on rural domestic waste management and avoid empty-headed policies. In this way, the policy needs of rural residents can be met, and their understanding of and confidence in the policy can be increased, thus motivating them to participate in domestic waste management.

## Data availability statement

The original contributions presented in the study are included in the article/supplementary material, further inquiries can be directed to the corresponding author.

## Author contributions

Conceptualization: XC, KW, and JL. Methodology and writing-original draft and editing: XC, LX, and JL. All authors were committed to improving this paper and are responsible for the viewpoints mentioned in this work.

## References

[1] Zhang B Lai KH Wang B Wang Z. From intention to action: How do personal attitudes, facilities accessibility, and government stimulus matter for household waste sorting? J Environ Manag. (2018) 233:447–58. 10.1016/j.jenvman.12059 30593004

[2] Stoeva K Alriksson S. Influence of recycling programmes on waste separation behavior. Waste, Management. (2017) 68:732–41. 10.1016/j.wasman.06005 28619237

[3] Manomaivibool P Srivichai M Unroj P Dokmaingam P. Chiang Rai zero waste: participatory action research to promote source separation in rural areas. Res Conserv Recycl. (2018) 136:142–52. 10.1016/j.resconrec.04002

[4] Nguyen TT Watanabe T. Win-win outcomes in waste separation behavior in the rural area: a case study in Vietnam. J Clean Prod. (2019) 230:488–498. 10.1016/j.jclepro.05120

[5] Aslanbeigui N Medema SG. Beyond the dark clouds: Pigou and Coase on social cost. History of Political, Economy. (1998) 30:601–25.

[6] Vicente P Marques C Reis E. Willingness to pay for environmental quality: the effects of pro-environmental behavior, perceived behavior control, environmental activism, and educational level. SAGE Open. 11:21582440211025256. 10.1177/21582440211025256

[7] Schultz PW Oskamp S Mainieri T. Who recycles and when? A review of personal and situational factors. J Environ Psychol. (1995) 15:105–21. 10.1016/0272-4944(95)90019-5

[8] Ma QZ Huang DJ Li H. Effects of fiscal decentralization on garbage classifications. Front Energy Res. (2022) 9:1–14. 10.3389/fenrg.2021.686561

[9] Stern PC Dietz T Kalof L. Values, beliefs, and proenvironmental action: attitude formation toward emergent attitude objects. J App Soc Psychol. (1995) 25:1611–36. 10.1111/j.1559-1995tb02636.x

[10] Zeng C Niu DJ Li HF. Public perceptions and economic values of source-collection of rural solid waste: a pilot study in China. Res Conserv Recycl. (2016) 107:166–73. 10.1016/j.resconrec.12010

[11] Wang Y Hao F. Public perception matters: individual waste sorting in Chinese communities. Res Conserv and Recycl. (2020) 159:1–12. 10.1016/j.resconrec.2020.104860

[12] Cecere G Mancinelli S Mazzanti M. Waste prevention and social preferences: the role of intrinsic and extrinsic motivations. Ecol Econ. (2014) 107:163–76. 10.1016/j.ecolecon.07007

[13] Xu L Ling ML Wu YL. Economic incentive and social influence to overcome household waste separation dilemma: a field intervention study. Waste Manag. (2018) 77:522–31. 10.1016/j.wasman.04048 29735360

[14] Zen IS Siwar C. An analysis of household acceptance of curbside recycling scheme in Kuala, Lumpur, Malaysia. Habitat Int. (2015) 47:248–55. 10.1016/j.habitatint.01014

[15] Liu Y Huang J. Rural domestic waste disposal: an empirical analysis in five provinces of China. China Agricult Econ Rev. (2014) 6:558–73. 10.1108/CAER-05-2013-0076

[16] Li X Bi F Han Z Qin Y Wang H Wu W. Garbage source classification performance, impact factor, and management strategy in rural areas of China: a case study in Hangzhou. Waste Manag. (2019) 89:313–21. 10.1016/j.wasman.04020 31079745

[17] Song YM Zhan YT Qi YB Xu DD Deng X. Does political participation influence the waste classification behavior of rural residents? Empirical evidence from rural China. Agriculture-Basel. (2022) 12:625. 10.3390/agriculture12050625

[18] Zhou SY Qing C Guo SL Deng X Song JH Xu DD. Why “Say One, Thing, and Do Another” a study on the contradiction between farmers,” intention and behavior of garbage classification. Agriculture-Basel. (2022) 12:1159. 10.3390/agriculture12081159

[19] Kosec K Wantchekon L. Can information improve rural governance and service delivery? World Develop. (2020) 125:104376. 10.1016/j.worlddev.07017

[20] Rijswijk K Klerkx L Bacco M. Digital transformation of agriculture and rural areas: a socio-cyber-physical system framework to support responsibilisation. J Rural Stud. (2021) 85:79–90. 10.1016/j.jrurstud.05003

[21] Qin TY Wang LJ Zhou YX. Digital technology-and-services-driven sustainable transformation of agriculture: cases of China and the, EU. Agricult Basel. (2022) 12:297. 10.3390/agriculture12020297

[22] Shen FW Chen XL. Maintaining rurality: a theoretical account of achieving the distinctive character of digital rural governance. E-*Government*. (2021) 39–48. Available online at: https://kns.cnki.net/kcms2/article/abstract?v=3uoqIhG8C44YLTlOAiTRKibYlV5Vjs7iy_Rpms2pqwbFRRUtoUImHepVaqcM361p4GmdFUlIb4aOv_VtbiJwLbJ9dHjODioanduniplatform=NZKPT (accessed October 2, 2022)

[23] Xia J. Linking ICTs to rural development: China's rural information policy. Govern Inform Quart. (2009) 27:187–95. 10.1016/j.giq.10005

[24] Cao L Niu H Wang Y. Utility analysis of digital villages to empower balanced urban-rural development based on the three-stage, DEA-, Malmquist model. PLoS ONE. (2022) 17:e0270952. 10.1371/journal.pone.0270952 35913937 PMC9342736

[25] Huang RL. Construction of rural governance digital driven by artificial intelligence and big Data. Math Prob Engin. (2022) 3:8145913. 10.1155/2022/8145913

[26] Cyberspace Administration of China (CAC). Digital Village Development Action Plan (2022-2025). Available online at: http://www.cac.gov.cn/2022-01/25/c_1644713313939252.htm

[27] Mergel I Edelmann N Haug N. Defining digital transformation: results from expert interviews. Govern Inform Quart. (2019) 36:101–385. 10.1016/j.giq.06002

[28] Zimmerman MA. Taking aim on empowerment research: on the distinction between individual and psychological conceptions. Am J Commun Psychol. (1990) 18:169–77.

[29] Han RB. Analysis of rural digital governance and its practice orientation from the perspective of agile governance. J South China Agricult Univ (Soc Sci Ed). (2021) 20:132–40. Available online at: https://kns.cnki.net/kcms2/article/abstract?v=3uoqIhG8C44YLTlOAiTRKibYlV5Vjs7iy_Rpms2pqwbFRRUtoUImHdpjsUQWZfBxtRL_Rc8xGtbwKdvzhoR3EZ7BsD-FTvH1&uniplatform=NZKPT (accessed October 18, 2022).

[30] China, Internet Network Information Center (CNNIC),.The 47th China Statistical Report on the Internet Development. Available online at: http://www.cac.gov.cn/2021-02/03/c_1613923423079314.htm

[31] Kil N Holland SM Stein TV. Structural relationships between environmental attitudes, recreational motivations, and environmentally responsible behaviors. J Outdoor Recreat Tour. (2014) 7:16–25. 10.1016/j.jort.09010

[32] Gamba RJ Oskamp S. Factors Influencing community resident's participation in commingled curbside recycling programs. Environ Behav. (1994) 26:587–612.

[33] López-Mosquera N Lera-López F Sánchez M. Key factors to explain recycling, car use and environmentally responsible purchase behaviors: a comparative perspective. Res Conserv Recycl. (2015) 99:29–39. 10.1016/j.resconrec.03007

[34] Padilla AJ Trujillo JC. Waste disposal and households' heterogeneity. Identifying factors shaping attitudes towards source-separated recycling in Bogotá, Colombia. Waste Manag. (2018) 74:16–33. 10.1016/j.wasman.11052 29258776

[35] Almasi A Mohammadi M Azizi A. Assessing the knowledge, attitude and practice of the Kermanshahi women towards reducing, recycling and reusing of municipal solid waste. Res Conserv Recycl. (2019) 141:329–38. 10.1016/j.resconrec.10017

[36] Banga M. Household knowledge, attitudes and practices in solid waste segregation and recycling: the case of urban Kampala. Zambia Soc Sci J. (2011) 2:4. Avalilable online at: https://scholarship.law.cornell.edu/zssj/vol2/iss1/4/ (accessed October 10, 2022).

[37] Gong XM Zhang JP Zhang HR Cheng M Wang F Yu N. Internet use encourages pro-environmental behavior: evidence from China. J Clean Prod. (2020) 256:120725. 10.1016/j.jclepro.2020.12072535085292

[38] Naldi L Nilsson P Westlund H Wixe S. What is smart rural development? J Rural Stud. (2015) 40:90–101. 10.1016/j.jrurstud.06006

[39] Arjomandi AP Yazdanpanah M Shirzad A Komendantova N Kameli E Hosseinzadeh M. Institutional trust and cognitive motivation toward water conservation in the face of an environmental disaster. Sustainability. (2023) 15:900. 10.3390/su15020900

[40] Huang YHC Lu YH Kao L. Mainframes and mandarins: The impact of internet use on institutional trust in East, Asia. Telecommun Policy. (2022) 44:101912. 10.1016/j.telpol.2020.101912

[41] Molin J Oberg-Nordin M Arvidsson B. A personal and professional journey-experiences of being trained online to be a supervisor in professional supervision in nursing. Int J Qualit Stud Health WellBeing. (2021) 16:1952523. 10.1080/17482021 34254902 PMC8279151

[42] Kathuria V. Informal regulation of pollution in a developing country: evidence from India. Ecol Econ. (2006) 63:425–36. 10.1016/j.ecolecon.1101312926702

[43] Zajonc RB. Social facilitation. Science. (1965) 149:269–74. 10.1126/science.149.3681.269 14300526

[44] National Development and Reform Commission (NDRC). Zhejiang Jiaxing Nanhu: Digital supervision system to help the new model of rural domestic waste separation. Available online at: https://www.ndrc.gov.cn/xwdt/ztzl/qgncggfwdxal/202204/t20220411_1321893.html?code=andstate=123

[45] Xu Q. “Micro-contact” and “Micro-autonomy”: the spatial extension and utility of modern rural social governance. J Huazhong Agricult Univ (Soc Sci Ed). (2020) 129–37+175. 10.13300/j.cnki.hnwkxb.2020.03.015

[46] Cao Y Zhang XL He LX. (2021). Collective Action in maintaining rural infrastructures: cadre-farmer relationship, institution rules and their interaction terms. Land Use Policy. 99:105043. 10.1016/j.landusepol.2020.105043

[47] Granovetter MS. Economic action and social structure: the problem of embeddedness. Am J Sociol. (1984) 19:481–510.

[48] Beugelsdijk S Schaik TV. (2004). Social capital and growth in European regions: an empirical test. Eur J Polit Econ. (2005) 21:301–24. 10.1016/j.ejpoleco.07004

[49] Zeng J Mao Y Xu M Jian B Qu M. Exploring the effect of individual and group level factors on the level of rural residents' domestic waste sorting: evidence from Shaanxi, China. Int J Environ Res Public Health. (2022) 19:12022. 10.3390/ijerph191912022 36231324 PMC9564579

[50] Luo H Zhao LG Zhang ZJ. The impacts of social interaction-based factors on household waste-related behaviors. Waste Manag. (2022) 118:270–80. 10.1016/j.wasman.08046 32919346

[51] Zobeidi T Komendantova N Yazdanpanah M. Social media as a driver of the use of renewable energy: the perceptions of instagram users in Iran. Energy Policy. (2022) 161:112721. 10.1016/j.enpol.2021.112721

[52] Xie JH Yang GQ Wang G Xia W. How do network embeddedness and environmental awareness affect farmers' participation in improving rural human settlements? Land. (2021) 10:1095. 10.3390/land10101095

[53] Munzner T. Visualization Analysis and Design. Boca Raton: CRC, Press (2015).

[54] Wang ZX. Institutional trust in East, Asia. in Asian Barometer Working Paper Series. (2013).

[55] Dalton RJ. Democratic, Challenges, Democratic Choices: The Erosion of Political Support in Advanced Industrial, Democracies. Oxford, New York: Oxford University Press (2004).

[56] Seligson MA Carrioán JF. Political support, political skepticism, and political stability in new democracies. Comparat Polit Stud. (2002) 35:58–82. 10.1177/001041400203500106

[57] Hetherington MJ Rudolph TJ. Why Washington Won't Work: Polarization, Political Trust, and the Governing Crisis. Chicago: University of Chicago Press. (2015).

[58] Li DG Jiang WJ Cai JJ. Individual norms and public action: how does institutional trust facilitate responsive public participation? a text data analysis based on urban water environment appeals. J Public Manag. (2022) 19:117–29. Available online at: https://kns.cnki.net/kcms2/article/abstract?v=3uoqIhG8C44YLTlOAiTRKibYlV5Vjs7iJTKGjg9uTdeTsOI_ra5_XcaV6U9otF18LxXbxuXMy0pj2SspEDUTD1JbQ_VtGs4e&uniplatform=NZKPT (accessed October 10, 2022).

[59] Sonia SMG. Trust, satisfaction, relational norms, opportunism and dependence as antecedents of employee organizational commitment. Contadurí*a Y Administ*. (2013) 58:11–38. Available online at: https://www.scielo.org.mx/scielo.php?script=sci_arttext&pid=S0186-10422013000200002 (accessed October 10, 2022).

[60] Herb S Hartmann E. Opportunism risk in service triads: a social capital perspective. Int J Phys Distrib Log Manag. (2014) 44:242–56. 10.1108/IJPDLM-08-2012-0249

[61] Zomeren MV Postmes T Spears R. Toward an integrative social identity model of collective action: a quantitative research synthesis of three socio-psychological perspectives. Psychol Bullet. (2008) 34:504–35. 10.1037/0033-1344.504 18605818

[62] He K Zhang JB Zeng YM. Rural households', willingness to accept compensation for energy utilization of crop straw in China. Energy. (2018) 165:562–71. 10.1016/jenergy09

[63] Taniguchi H Marshall GA. Trust, political orientation, and environmental behavior. Environ Polit. (2018) 27:385–410. 10.1080/09644016.2018.1425275

[64] Jia YJ Cheng SJ Shi R. Decision-making behavior of rural residents', domestic waste classification in Northwestern of China—Analysis based on environmental responsibility and pollution perception. J Clean Prod. (2021) 326:129374. 10.1016/j.jclepro.2021.129374

[65] He LX Zhang ZG Nan YQ Lin JY. Institutional rules and cadre-farmer relationship: solve the dilemma in rural infrastructures' maintenance action. Issues Agricult Econ. (2017) 38:9–21+110. https://kns.cnki.net/kcms2/article/abstract?v=3uoqIhG8C44YLTlOAiTRKibYlV5Vjs7iAEhECQAQ9aTiC5BjCgn0RkSEPUQPkRy688XJk0dd361z6KMdnlPUM9buKv2DcqDG&uniplatform=NZKPT (accessed October 10, 2022)

[66] Roodman D. Fitting fully observed recursive mixed-process models with, CMP. Stata J. (2011) 11:159–206. 10.1177/1536867X1101100202

